# Honey as a Potential Natural Antioxidant Medicine: An Insight into Its Molecular Mechanisms of Action

**DOI:** 10.1155/2018/8367846

**Published:** 2018-01-18

**Authors:** Sarfraz Ahmed, Siti Amrah Sulaiman, Atif Amin Baig, Muhammad Ibrahim, Sana Liaqat, Saira Fatima, Sadia Jabeen, Nighat Shamim, Nor Hayati Othman

**Affiliations:** ^1^Department of Pathology, School of Medical Sciences, Universiti Sains Malaysia, Kubang Kerian, 16150 Kelantan, Malaysia; ^2^Department of Biochemistry, Bahauddin Zakariya University, Multan 60800, Pakistan; ^3^Department of Pharmacology, School of Medical Sciences, Universiti Sains Malaysia, Kubang Kerian, 16150 Kelantan, Malaysia; ^4^Faculty of Medicine, Universiti Sultan Zainal Abidin, Darul Iman, Kuala Terengganu, 20400 Terengganu, Malaysia

## Abstract

Honey clasps several medicinal and health effects as a natural food supplement. It has been established as a potential therapeutic antioxidant agent for various biodiverse ailments. Data report that it exhibits strong wound healing, antibacterial, anti-inflammatory, antifungal, antiviral, and antidiabetic effects. It also retains immunomodulatory, estrogenic regulatory, antimutagenic, anticancer, and numerous other vigor effects. Data also show that honey, as a conventional therapy, might be a novel antioxidant to abate many of the diseases directly or indirectly associated with oxidative stress. In this review, these wholesome effects have been thoroughly reviewed to underscore the mode of action of honey exploring various possible mechanisms. Evidence-based research intends that honey acts through a modulatory road of multiple signaling pathways and molecular targets. This road contemplates through various pathways such as induction of caspases in apoptosis; stimulation of TNF-*α*, IL-1*β*, IFN-*γ*, IFNGR1, and p53; inhibition of cell proliferation and cell cycle arrest; inhibition of lipoprotein oxidation, IL-1, IL-10, COX-2, and LOXs; and modulation of other diverse targets. The review highlights the research done as well as the apertures to be investigated. The literature suggests that honey administered alone or as adjuvant therapy might be a potential natural antioxidant medicinal agent warranting further experimental and clinical research.

## 1. Introduction

Current stream treatment modalities utilizing chemo drugs dissimulate multidrug resistance and several other side effects [1]. This urges to quest for alternate options. Natural products are pondered as a practical alternative approach to abate the ever increasing scold of diseases and some of their unavoidable side effects [2, 3]. Recently, honey as a natural product has clinched the attention of researchers as a complementary and alternative medicine [4–6].

**Figure 1:**
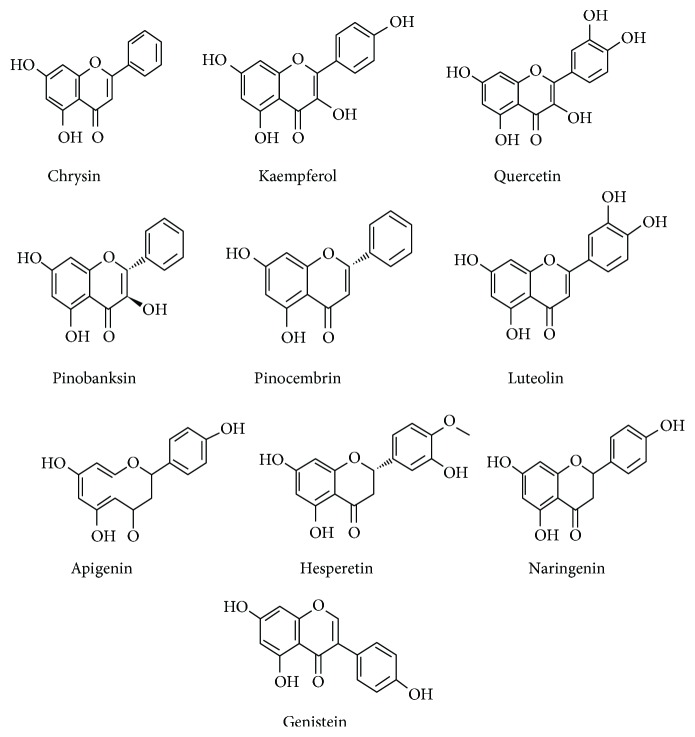
Chemical structures of flavonoids in honey [17].

**Figure 2:**
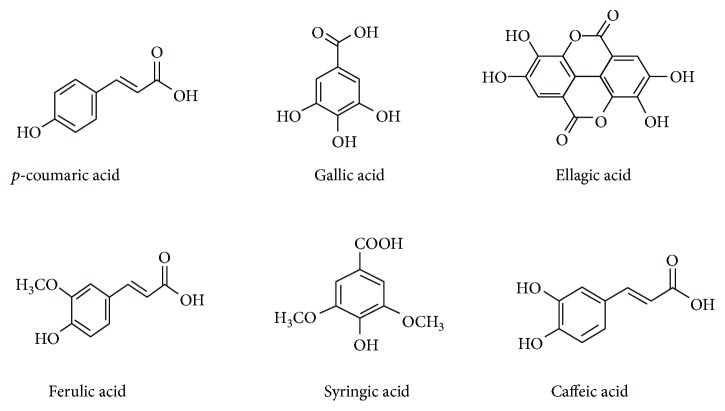
Chemical structures of phenolic acids in honey [17].

Honey as a folk medicine is referred in the utmost ancient written archives [7, 8]. Demarcation of its uses in current professional medicine as a potential therapy is entirely underutilized. However, there is an affinity for some researchers to fire out a coherent proposition that usage of honey as a natural product supplement is well intentioned for reflection as a therapy or adjuvant antioxidant therapy in current medicine [9, 10]. The composition of honey varies from floral source to origin. A general average composition of honey has been presented in Table 1. It is composed of at least 181 substances and primarily fabricates the fructose (38%) and glucose (31%) as major sugars. Besides fructose and glucose, other identified disaccharides include maltose, sucrose, maltulose, turanose, isomaltose, laminaribiose, nigerose, kojibiose, gentiobiose, and B-trehalose. Trisaccharides include maltotriose, erlose, melezitose, centose 3-a5, isomaltosylglucose, l-kestose, isomaltotriose, panose, isopanose, and theanderose [11]. It also comprises enzymes, amino acids, proteins, flavonoids, phenolic acids, and a miscellaneous group. There are 26 amino acids reported in honey; among them, proline is the major contributor that constitutes 50–85% of the total amino acids [12]. The minor volume of vitamins includes riboflavin, niacin, folic acid, pantothenic acid, vitamin B6, and ascorbic acid. Different trace elements cover calcium, iron, zinc, potassium, phosphorus, magnesium, selenium, chromium, and manganese. Organic acids are other important group of compounds in honey, for instance, acetic, butyric, citric, succinic, lactic, malic, and gluconic acid and a number of other aromatic acids [13]. The various enzymes present in honey are glucose oxidase, sucrose diastase, catalase, and acid phosphatase [14–16]. Some of the flavonoids and phenolic compounds that have been identified in honey include kaempferol, quercetin, chrysin, pinobanksin, luteolin, apigenin, pinocembrin, genistein, hesperetin, *p*-coumaric acid, naringenin, gallic acid, ferulic acid, ellagic acid, syringic acid, vanillic acid, and caffeic acid [17, 18]. Flavonoids and phenolic acid constituents have been reported to be solely responsible for the antioxidant and other medicinal effects of honey [6, 18–24]. The chemical structures of major flavonoids and phenolic acids in honey have been demonstrated in Figures 1 and 2.

Honey has been studied against various ailments in animal and human models. Published research denotes it as a novel antioxidant agent [24, 25]. It exhibits a broad spectrum therapeutic properties such as anti-inflammatory [26], antibacterial [27], antimutagenic [28], expedite wound healings [29], antidiabetic [30], antiviral [31], antifungal [32], and antitumoural [5, 33, 34] effects. It could be purported as a natural cancer “vaccine” as it reduces chronic inflammation, improves healing of chronic ulcers and wounds, and improves immune status; the opposite of these are risk factors to cancer formation [5]. Its anticancer activity has been proved against various types of cancer: breast [35–39], colorectal [40], renal [41], prostate [36], endometrial [36], cervical [39], and oral [42]. Honey has the potential to reduce cardiovascular risk factors in normal healthy individuals [43]. It causes to reduce systolic blood pressure and level of triglycerides and VLDL (low-density lipoprotein) in experimental animals [44]. In a randomized clinical trial, lower incidence of acute respiratory symptoms was observed in individuals who took honey on a daily basis [45]. It improves female hormones [46], increases the percentage of sperms and motility, and reduces the toxic effects on spermatogenesis and testosterone level [47, 48]. Postmenopausal women who received honey therapy showed improvement in their immediate memory compared with the improvement seen in women receiving estrogen plus progestin therapy [49].

Understanding the mode of action of honey is substantial and under phase area. The review presents a role of honey in modulation of different types of diseases and the possible mechanisms involved. It also highlights a synopsis of findings through which it makes a road from different signaling pathways to different molecular targets. The review also shows the rational explanations for the therapeutic effects of honey and the apertures to be investigated.

## 2. Medicinal Effects of Honey and Mechanisms of Action

### 2.1. Antioxidant Effects of Honey

**Figure 3:**
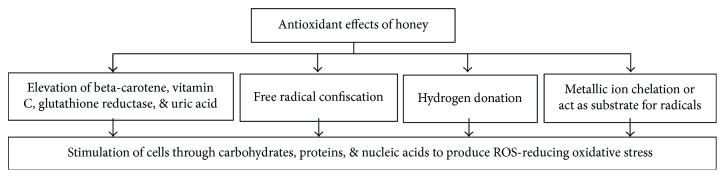
Mechanisms of antioxidant effects of honey.

Antioxidants are agents to counteract deterioration caused by oxidants such as such as O_2_, OH^−^, superoxide, and/or lipid peroxyl radicals. Cancer, synthesis of mutagens, aging, atherosclerosis, and many chronic and degenerative lingering diseases are susceptible to oxidative stress [52]. Cells exhibit defense system against oxidative damage. This defense system consists of free radicals and other oxidative protective agents such as, catalase, superoxide dismutase, peroxidase, ascorbic acid, tocopherol, and polyphenols [53]. These antioxidant agents stimulate biomolecules such as carbohydrates, proteins, lipids, and nucleic acids. Cells are altered by this stimulation and ultimately provoking antioxidant response [54]. Honey exhibits strong antioxidant activity [6]. This antioxidant capacity of honey contributes to the prevention of several acute and chronic disorders such as inflammatory, allergic, thrombotic, diabetic, cardiovascular, cancer, and others. The antioxidant properties of honey can be measured in the form of antiradical activity using, oxygen radical absorbance capacity (ORAC) assay, 1,1-diphenyl-2-picrylhydrazyl (DPPH) scavenging assay, and ferric reducing antioxidant power (FRAP) assay [24]. Honey from various floral origin and different countries has been shown to exhibit high antioxidant properties [24]. The phenolic acids and flavonoids are responsible for the well-established antioxidant activity of honey. Apart from these, sugars, proteins, amino acids, carotenes, organic acids, Maillard reaction products, production of reactive oxygen species (ROS), and other minor components also contribute to antioxidant effect [53, 55]. Researchers also showed that honey (1.2 g/kg) elevated the amount and activity of antioxidant agents such as beta-carotene, vitamin C, glutathione reductase, and uric acid in healthy human subjects [56]. The exact antioxidant mechanism is unknown, but the proposed mechanisms include free radical sequestration, hydrogen donation, metallic ion chelation, flavonoids substrate action for hydroxyl, and superoxide radical actions [25, 57].  Figure 3 is presenting all the possible mechanisms involved in the antioxidant effects of honey. The antioxidant effect of honey is well established, but urges to explore the exact mechanisms involved and extrapolation to clinical trials.

### 2.2. Antibacterial and Wound Healing Effects of Honey

Different clinical trials and in vitro studies have reported broad-spectrum antimicrobial properties of honey [58]. It was reported that honey constrains the growth of pathogenic strains such as *Streptococcus pyogenes, Streptococcus typhi*, *Staphylococcus aureus*, coagulase-negative *Streptococcus* and *E.coli*, and species [59]. It also diminishes the growth of infecting strains such as *Pseudomonas aeruginosa, Acinetobacter baumannii*, and *Klebsiella pneumonia* in full thickness burn wound in rats [60].

Antibacterial effect of honey is attributed to presence of inert antibiotic factors in it. These factors include its acidic pH, osmotic effect of sugars, and production of H_2_O_2_ by peroxidase. Some nonperoxidase substances also support antibacterial activity which include flavonoids, phenolic acids, and lysozyme [61]. In its mechanism of action, a significant role is played by bee defensin-1(antimicrobial peptide), methylglyoxal (phytochemical), and hydrogen peroxide production by enzyme glucose oxidase [62]. Furthermore, high sugar contents of honey can also be helpful in eliminating bacteria through osmosis [63]. Methylglyoxal (MGO) in honey and its precursor dihydroxyacetone (DHA) have been recognized as inhibitors of bacterial growth through urease inhibition. Urease enzyme facilitates bacteria to acclimate and grow rapidly by producing ammonia in acidic environment [64]. A very recent study reveals that honey combats bacterial infections by two different mechanisms: inhibition of bacterial quorum sensing (QS) system to retard the expression of *las*, *MvfR*, and *rhl* regulons, as well as its associated virulence factors, and bactericidal components which actively kill bacterial cells [65].

Biofilms have emerged as a key factor in antibiotic resistance. Biofilms protect bacteria from antibiotics resulting in relentless infection. Honey acts as a bactericidal negotiator, penetrates in biofilms, recovers aggressive infection, and eradicates colonies [66, 67]. It has shown bactericidal effect against biofilms of pathogenic reference strains such as *Methicillin-resistant Staphylococcus epidermidis* (MRSE), *Extended-spectrum beta-lactamases* (ESBL), *Klebsiella pneumonia*, *Pseudomonas aeruginosa*, *Staphylococcus aureus* (SA), *Proteus mirabilis*, *Pseudomonas aeruginosa* (PA), *Clostridium difficile*, and *enterohemorrhagic E. coli*. It improves wounds healing, prevents invasive infections, eliminates biofilm colonization, interrupts outbreaks, and thus preserves current antibiotic stocks [66, 68–70]. It inhibits biofilm growth by preventing the binding of bacterial strains with tissue fibronectin at infection site. It also reduces expression of fibronectin binding surface proteins such as Sfb1 and Sof, which are crucial for bacteria to bind with fibronectin [71]. It also significantly suppresses the expression of quorum sensing genes (AI-2 importer and indole biosynthesis), curli genes (csgBAC), and virulence genes (LEE genes) in virulent *E. coli*. Glucose and fructose content in the honey were considered to be key components in repressing biofilm formation [72].

Normal wound healing is a multipart process in which coinciding series of events occur which include coagulation, inflammation, cell proliferation, tissue remodeling, and replacement of damaged tissue [73]. Honey has been used widely for the treatment of various types of chronic, burn, necrotic, diabetic foot and postoperative split skin wounds [61, 74–76]. In inflammatory phase of wound healing, honey assists in the elimination of necrotic tissues [63], improves the remodeling phase [63], and inhibits bacterial growth [59], resulting in improved healing. Recent study indicates an increased production of IL-6 and TNF-*α* by honey at the wound site in the healing process in IL-6-deficient mice [77]. Honey facilitates an increased stimulation and production of lymphocytes, phagocytes, monocytes, and/or macrophages to release cytokines and interleukins such as TNF-*α*, IL-1*β*, and IL-6, expediting the healing process [78]. High sugar contents and osmolarity of honey also contribute towards healing. Water is drawn out from the wound bed by the osmotic effect of honey through a simple outflow of lymph if the blood circulation at the wound site is sufficient to carry out this process [79]. Research has shown that honey improves wound healing through antioxidant response by activating AMPK (5′adenosine monophosphate-activated protein kinase) and antioxidant enzymes which ameliorate oxidative stress. The antioxidant system comprises exogenous and endogenous antioxidants. The endogenous antioxidants are classified as enzymatic and nonenzymatic antioxidants. The enzymatic antioxidants include superoxide dismutase (SOD), catalase (CAT), and glutathione peroxidase (GPx). The nonenzymatic antioxidants comprise vitamins E and C, glutathione (GSH), and some small molecules, while exogenous antioxidants include some micronutrients [24, 80]. These antioxidants also support proliferation and migration of human dermal fibroblasts and mitochondrial function to assist healing [81].

Another mechanism explains that wound sites have usually two types of protein-digesting enzymes: serine proteases and matrix metalloproteases. These protease enzymes are generally inactive due the presence of some inhibitors. The proteases become active when the inhibitors become inactive by H_2_O_2_. Thus, H_2_O_2_ plays a role as physiological switching stimuli for activation and inactivation of these enzymes through oxidation. It has been reported that honey stimulates and enhances H_2_O_2_ production. The wound debris and bacteria are digested by active proteases. The active effect of honey sweeps off this debris easily due to the osmotic outflow [7, 82]. During inflammation, H_2_O_2_ also stimulates the growth of fibroblasts and epithelial cells to repair the damage. Similarly, H_2_O_2_ stimulates nuclear transcription factors (NTFs) for cell multiplication and wound healing [7].

**Figure 4:**
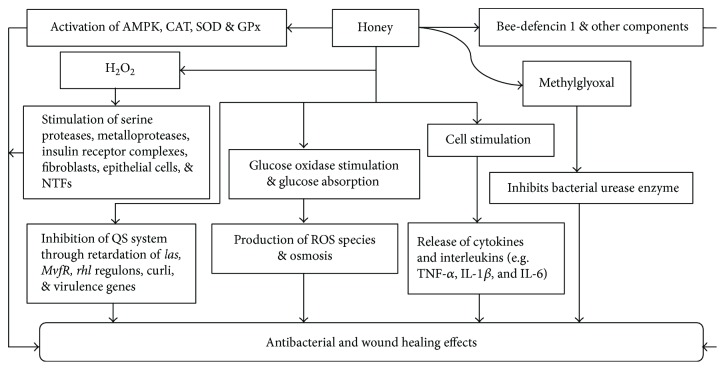
Mechanisms of antibacterial and wound healing effects of honey. AMPK = 5′adenosine monophosphate-activated protein kinase; QS = quorum sensing; SOD = superoxide dismutase; GPx = glutathione peroxidase; NTFs = nuclear transcription factors; TNF-*α* = tumour necrosis factor alpha; IL = interleukins.

Some additional mechanisms elaborate that H_2_O_2_ stimulates insulin receptor complexes to trigger a chain of molecular events in the cell. This results in facilitating the uptake of amino acids and glucose for cell growth. Honey itself may provide vitamins, minerals, sugars, and amino acids to the growing cells. This supports phagocytes to engulf infecting bacteria through glucose consumption. Honey also stimulates cytokines release from monocyte and lymphocyte proliferation to repair tissues. Monocyte activation by mitogen or honey leads to the production of reactive oxygen species to initiate a greater inflammatory response. It causes oedema in surrounding tissue restricting circulation in capillaries. This results in reduction of oxygen supply and nutrients to cells. It ultimately restricts the cell growth to replace tissues to repair wounds [7, 83]. All the possible mechanisms involved in antibacterial and wound healing effects of honey have been demonstrated in Figure 4.

### 2.3. Antifungal Effects of Honey

Honey exhibits antifungal activity. Research has shown that it has antifungal activity against *Aspergillus niger*, *Aspergillus flavus*, *Penicillium chrysogenum*, *Microsporum gypseum*, *Candida albicans*, *Saccharomyces*, and *Malassezia* species [84]. The potential antimicrobial effect of honey is attributed to the presence of glucose oxidase, methylglyoxal, and high sugar contents [85–88]. The mechanism is not completely understood; however, some potential pathways have been suggested.

**Figure 5:**
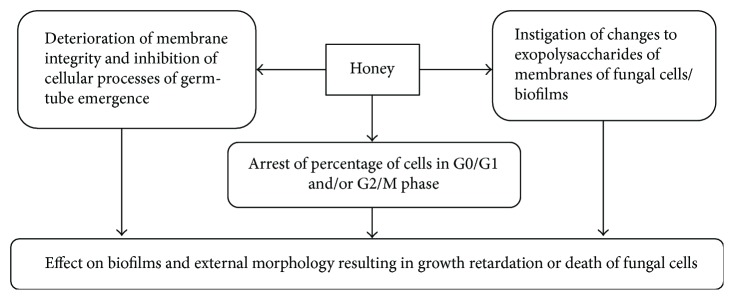
Mechanisms of antifungal effects of honey.

Honey inhibits fungal growth through prevention of their biofilm formation, disruption of established biofilms, and instigating changes to exopolysaccharide structure. It distorts the cell membrane integrity which results in shrinkage of cell surface in biofilm, leading to death or growth retardation [89]. Atomic force microscopic studies have revealed that when biofilm is treated with honey (40% *w*/*v*) exopolysaccharide layer thickness is reduced to half and roughness increases followed by its complete removal [90]. Researchers have shown that flavonoid part of honey decelerates the growth of fungi, affects the external morphology and membrane integrity, and inhibits some cellular processes that are involved in germ-tube growth. The inhibition of germ-tube emergence correlates with poor growth of membrane. Honey flavonoid extract has also been found to affect hyphal transition by reducing the percentage of cells in the G0/G1 phase and/or G2/M phase [91].  Figure 5 is showing the possible mechanisms involved in antifungal effects of honey. The detailed description of antifungal effects of honey and molecular targets involved is a key gap to be probed yet.

### 2.4. Antiviral Effects of Honey

**Figure 6:**
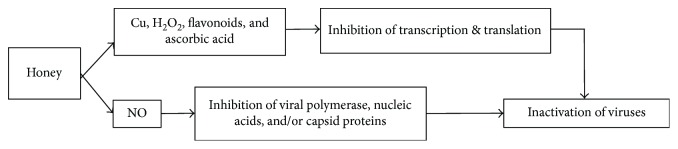
Mechanisms of antiviral effects of honey. Cu = copper; NO = nitric oxide.

The viral activity is usually elicited by native or universal stimuli which lead to infections and lesions [92]. Current studies have manifested that honey holds potential antiviral effects. Antiviral effect of honey is attributed to its various ingredients which have been found to be operative in controlling of lesions, for instance, copper inactivates virus that is a trace element part of honey. Similarly, presence of ascorbic acid, flavonoids, and H_2_O_2_ production by honey also leads to viral growth inhibition by interrupting viral transcription and translation [93, 94]. Data of in vitro studies has shown antiviral activity of honey against different types of viruses such as *Rubella*, *herpes simplex*, and *varicella zoster* viruses [31, 95, 96]. Honey comprises secretion from the salivary and pharyngeal glands of the honeybee's head. Recently, nitric oxide (NO) metabolites, nitrite, and nitrate have been identified in salivary gland's section [56]. It is well established that NO is an energetic molecule that produces host defense against viruses, both DNA and RNA viruses. NO acts by slowing down the development of viral lesions as well as arresting their replication [56, 97]. In its mode of action, NO represses replication by interfering with viral polymerase, nucleic acid, and/or viral capsid proteins. The flavonoid content of honey has also been reported to inhibit the viral transcription and replication [98, 99].  Figure 6 is presenting the possible mechanisms involved in antiviral activity of honey. To understand the actual influence of honey on viruses and mechanisms intends to do more research to map the road.

### 2.5. Anti-Inflammatory Effects of Honey

Inflammation is the intricate biological response of vascular tissues to detrimental stimuli. It is a defensive way of response shown by the tissues and organism to remove the pathogens or stimuli which are the cause of injury. Inflammation is classified into two classes: acute and chronic inflammation. Acute inflammation is an early retort of the body towards stimuli. The indication of acute inflammation is redness, pain, itching, and loss of ability to perform function [100]. If the acute inflammation is not treated well and prolonged, then it is converted into chronic inflammation. It is considered as a major cause of several chronic diseases or disorders. Thus, anti-inflammatory action is supposed to counteract unceasing diseases such as liver diseases [101], kidney diseases [102], and cancer [103]. Several factors can be involved in proinflammatory response such as cytokines, cyclooxygenases (COXs), lipoxygenases (LOXs), mitogens, macrophages, TNF factors, and many other factors of inflammatory pathways.

The anti-inflammatory action of honey is well documented [104]. It has shown anti-inflammatory response from cell cultures [40], animal models, to clinical trials [104, 105]. The exact mechanism of action of honey towards inflammation is not well understood yet. In inflammatory pathway, two of its components activated in ailments are mitogen-activated protein kinase (MAPK) and nuclear factor kappa B (NF-*κ*B) pathways [120]. Activation of MAPK and NF-*κ*B ultimately results in induction of several other inflammatory mediators, enzymes, cytokines, proteins, and genes such as cyclooxygenase-2 (COX-2), lipoxygenase 2 (LOX-2), C-reactive protein (CRP), interleukins (IL-1, IL-6, and IL-10), and TNF-*α*. All these markers of proinflammatory action are known to play a major role in inflammation and angiogenesis-related etiology of disease [30, 119]. Recent evidence of *in vivo* studies has shown the anti-inflammatory mechanisms of honey. These studies showed that honey decreases edema and plasma levels of proinflammatory cytokines such as IL-6, TNF-*α*, PGE2, NO, iNOS, and COX-2. It was also demonstrated that honey attenuates NF-*κ*B translocation to the nucleus and suppresses I*κ*B*α* (inhibitor of kappa B) degradation [106, 107]. It has been reported that phenolic acids and flavonoids such as chrysin, quercetin, and galangin are able to suppress the activity of proinflammatory enzymes, for example, cyclooxygenase-2 (COX-2), prostaglandins [108], and inducible nitric oxide synthase (iNOs) [109]. Research has shown that flavonoid content of honey slows down the expression of MMP-9 (matrix metallopeptidase 9), an inflammatory mediator that causes chronic inflammation. Honey has the ability to significantly inhibit the expression of anti-inflammatory cytokines such as IL-1 and IL-10 and growth factors PDGF (platelet-derived growth factor) and TGF-*β* (transforming growth factor-*β*). In vitro model of MM6 cell lines using 1% solution of honey was concluded [110]. Another possible mechanism shows that reactive oxygen species are produced by macrophages, monocytes, and neutrophils that enhance inflammation. Honey ceases the release of such types of cells to promote anti-inflammatory effect. It also inhibits the production of keratinocytes and leukocytes to reduce inflammation. It has been demonstrated that in inflammatory response H_2_O_2_ production by honey stimulates the growth of fibroblasts and epithelial cells to repair the inflammatory damage. This anti-inflammatory action of honey makes it a novel agent to modulate a disease [111–113].

**Figure 7:**
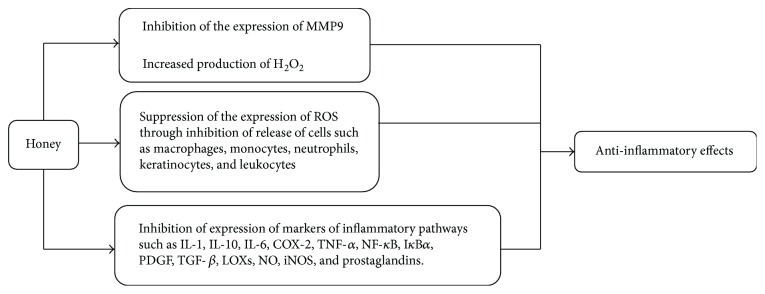
Mechanisms of anti-inflammatory effects of honey. MMP-9 = matrix metallopeptidase 9; IL = interleukin; COX-2 = cyclooxygenase 2; LOXs = lipoxygenases; TNF-*α* = tumour necrosis factor alpha; PGE2 = prostaglandin E2; NO = nitric oxide; iNOS = inducible nitric oxide synthase; NF-*κ*B = nuclear factor kappa B; I*κ*B*α* = inhibitor of kappa B; PDGF = platelet-derived growth factor; TGF-*β* = transforming growth factor-*β*.

Anomalous arachidonic acid metabolism is involved in inflammation. LOXs metabolize arachidonic acid to leukotrienes (LTs). There are three types of lipoxygenase (LOX) isozymes: 12-LOX, 15-LOX, and 5-LOX. 12-LOX provokes inflammatory/allergic disorders, 15-LOX synthesizes anti-inflammatory 15-HETE (15-hydroxyeicosatetraenoic acid), and 5-LOX generates 5-HETE (5-hydroxyeicosatetraenoic acid) and LTs [114]. Many polyphenols in honey have been reported to suppress LOXs [114]. The anti-inflammatory effect of honey can be attributed to its phenolic compounds and flavonoids [15, 128, 129].  Figure 7 is depicting the possible mechanisms of anti-inflammatory effect of honey. To understand the actual influence of honey on LOXs, COXs, and TNF signaling pathways and mechanisms involved intends to do more research to map the road.

### 2.6. Honey and Its Antidiabetic Properties

Diabetes mellitus is a complex metabolic syndrome. Insulin deficiency or nonfunctional insulin is responsible for it [115]. In this syndrome, many anomalies in lipoprotein and carbohydrate metabolism are involved with an elevated glucose level [116, 117]. Acute complications in this disorder may include hyperosmolar, diabetic ketoacidosis and hyperglycemic state, which may lead to death [118].

Honey has shown antidiabetic effects from animal models to clinical trials [30, 119]. Researchers have invoked it as a potential antidiabetic agent [120]. Its concentrations tested such as 0.2, 1.2, and 2.4 g/kg/day showed an improved antioxidant effect exerting a hypoglycemic in streptozotocin-induced diabetic rats [30]. Similarly, glucose level in type-2 diabetes mellitus was found to be reduced when honey was administered by inhalation as 60% (*W*/*V*) [119]. This antidiabetic or hypoglycemic effect of honey is attributed to the presence of fructose in it [122]. Fructose assists to regulate the insulin-response system, resulting in controlled blood glucose level. Another hypothesis suggests that glucose level is reduced by the postponement of digestion and absorption which are brought about by oligosaccharide palatinose, a sucrose. It results in modulation of diabetes in diabetic patients [123]. It has also been reported that captivation of glucose in cells can be increased in collaboration with fructose [124, 125], leading to a decreased food-intake or absorption to direct a hypoglycemic effect. Monosaccharides such as glucose, fructose, and galactose are formed by the hydrolysis of carbohydrates prior to their absorption [126]. It has been suggested that fructose is taken up by the two receptors GLUT5 and/or GLUT2 via protein- and energy-mediated diffusion [127]. The expression of GLUT2 mRNA is generally increased by glucose and fructose. However, an increased expression of GLUT5 mRNA is caused solely by fructose, resulting in its fast absorption [128–130]. Research has shown that a hypoglycemic effect was observed when mice induced with diabetes were fed with fructose [131]. Glucose level can also be regulated by a specific hypoglycemic role of the fructose in the liver. In this mode of action, fructose stimulates the phosphorylation enzymes, for instance, glucokinase, triggering hepatic glucose phosphorylation [132]. The inhibition of these enzymes results in inhibition of glycogenolysis. Thus, whole metabolism of glycogen and glucose is regulated by fructose, showing its vital regulatory role to control hyperglycemia [133, 134].

Another proposed mechanism explains that hypoglycemic effect of honey may be through the role of honey in modulating the insulin signaling pathway [120, 135]. A key component of insulin signaling is the PI3K/Akt [136]. It is known for its role in modulatory functions of several substrates which regulate cell cycle progression, cell survival, and cellular growth. The effect of honey extracts on Akt-activated insulin signaling pathway in pancreatic cells was recently investigated under hyperglycemic condition. It was observed that the development of insulin resistance was characterized by increased levels of NF-*κ*B, MAPK, and insulin receptor substrate 1 (IRS-1) serine phosphorylation. Akt expression and insulin contents were found to be markedly reduced. This study showed that pretreatment with honey and quercetin extract improves insulin resistance and insulin contents. Honey treatment increased the expression of Akt and reduced the expression of IRS-1 serine phosphorylation, NF-*κ*B, and MAPK [120, 135–137].

**Figure 8:**
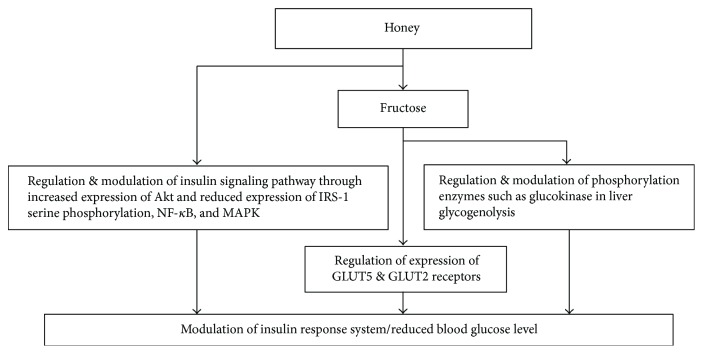
Mechanisms of antidiabetic effects of honey. MAPK = mitogen-activated protein kinase; NF-*κ*B = nuclear factor kappa B; Akt = altered PI3 kinase; IRS-1 = insulin receptor substrate 1.

Honey supplementation shows its modulatory effects on oxidative stress and hyperglycemia. Its antioxidant activity to ameliorate diabetes is well established [24]. Besides this, it also ameliorates several other metabolic derangements observed in diabetes such as reduced levels of triglycerides, hepatic transaminases, glycosylated hemoglobin (HbA1c), and increased HDL cholesterol [138].  Figure 8 is showing the possible mechanisms of antidiabetic effects of honey. Further studies are warranted to explore the exact mechanisms involved in antidiabetic activity of honey.

### 2.7. Antimutagenic Effects of Honey

**Figure 9:**
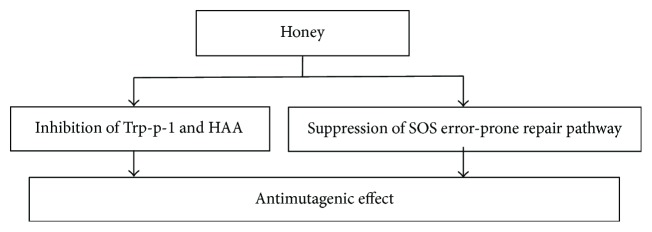
Mechanisms of antimutagenic effects of honey. Trp = tryptophan; HAA = heterocyclic aromatic amines; SOS = response of an error-prone repair pathway contributing to mutagenicity.

Mutagenicity, the ability to induce genetic mutation, is interlinked with carcinogenicity [139]. Honey exhibits strong antimutagenic activity [140]. The effect of honey on radiation (UV or *γ*)-exposed *Escherichia coli* cells was investigated to observe SOS response, which is an error-prone repair pathway contributing to mutagenicity [140]. Some important genes such as *umuC*, *recA*, and *umuD* involved in SOS-mediated mutagenesis were knocked out to elaborate the results. Honey reduced mutation frequency significantly in treatment groups than in controls. The suppression of error-prone mutagenic repair pathways (for instance SOS response in *E. coli*) was the possible mechanism contributing to the antimutagenic effect. The antimutagenic activity of honey from seven different floral sources (acacia, buckwheat, fireweed, soybean, tupelo, and Christmas berry) and honey sugar analogue against Trp-p-1 was tested by the Ames assay [28]. All honeys showed a significant inhibition of mutagenicity caused by Trp-p-1. About 30% honey in the infusion formulation was most effective in inhibiting HAA (heterocyclic aromatic amines) formation and overall mutagenicity beef steak and chicken breast [141].  Figure 9 is showing the possible mechanisms of antimutagenic effects of honey. A broad spectrum research is needed to conduct to understand the mechanisms of antimutagenic effects of honey.

### 2.8. Anticancer Effects of Honey

Cancer cells possess two distinct characteristics: unrestrained cell multiplication and inadequate apoptotic turnover [142]. Drugs which are commonly used for cancer treatment are apoptosis inducers [143]. Programmed cell death or apoptosis is categorized into three phases: (a) an induction phase, (b) an effector phase, and (c) a degradation phase. The induction phase stimulates proapoptotic signal transduction cascades through death-inducing signals (ceramide signaling, reactive oxygen species, Bcl-2 family proteins such as Bad, Bax, and Bid, and over activation of Ca^2+^ signalling pathway). Effector phase is committed to bring cell death via a key regulator, mitochondrion. The last degradation phase comprises nuclear and cytoplasmic events. Nuclear change includes chromatin and nuclear condensation, cell shrinkage, DNA fragmentation, and membrane blebbing. In the cytoplasm, a complex cascade of protein-cleaving enzymes called caspases is activated. The cell is finally destined into fragmented apoptotic bodies, which are phagocytosed by macrophages or other surrounding cells [143, 144].

The apoptosis usually follows two pathways: the caspase-8 or death-receptor pathway and caspase-9 or mitochondrial pathway.

Literature established that honey induces apoptosis in various types of cancer cells [22, 39, 40, 145, 146]. This apoptotic temper of honey is vital because many drugs used for cancer treatment are apoptosis inducers [147]. Thus, the honey and its active components can regulate apoptosis by operating at various points of these two signaling pathways.

**Figure 10:**
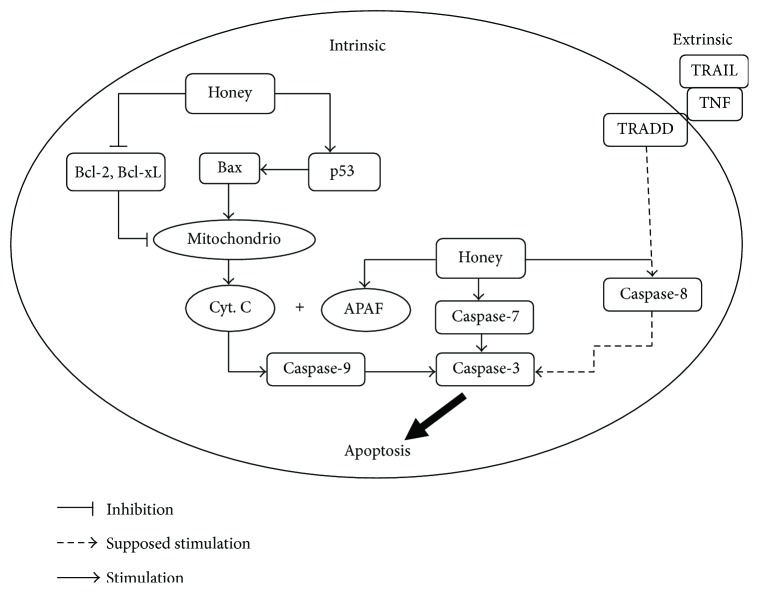
Effect of honey on apoptotic pathways (adopted from [6]). Bcl-2 = B cell lymphoma 2; Bcl-xL = B cell lymphoma extra large; Cyt. C = cytochrome C; APAF-1 = apoptotic protease activating factor 1; TNF = tumor necrosis factor; TRAIL = TNF-related apoptosis-inducing ligand; TRADD = TNFR-associated death domain protein.

**Figure 11:**
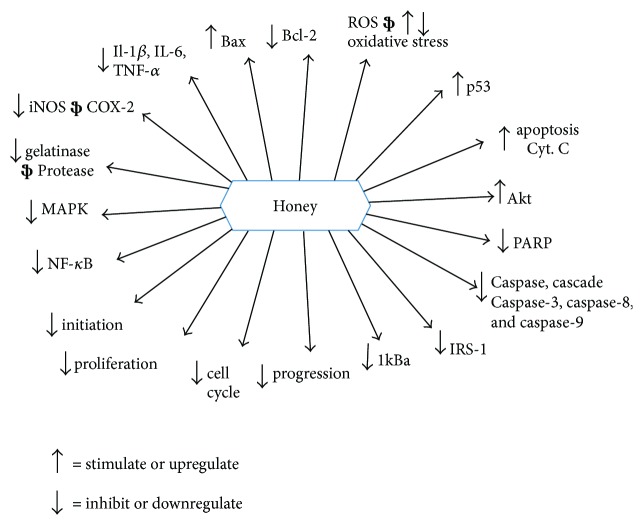
Molecular target modulation—the anticancer effects of honey (adopted from [17]). Bcl-2 = B cell lymphoma 2; Bcl-xL = B cell lymphoma extra large; Cyt. C = cytochrome C; MAPK = mitogen-activated protein kinase; NF-*κ*B = nuclear factor kappa B; Akt = altered PI3 kinase; IRS-1 = insulin receptor substrate; IL = interleukin; COX-2 = cyclooxygenase 2; TNF-*α* = tumour necrosis factor alpha; iNOS = inducible nitric oxide synthase; I*κ*B*α* = inhibitor of kappa B; PARP = poly ADP-ribose polymerase.

**Figure 12:**
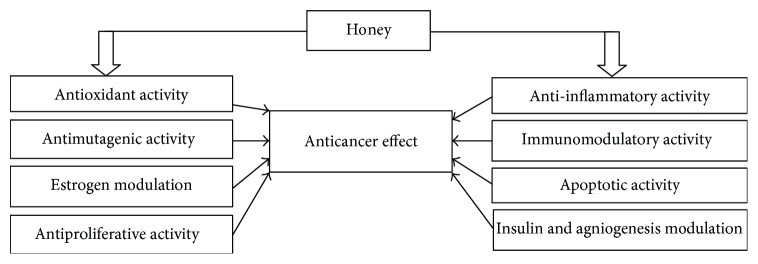
Schematic summary of anticancer effects of honey (adopted from [6]).

Honey induces apoptosis in human breast, colon, and cervical cancer cell lines model via depolarization of the mitochondrial membrane by reducing the mitochondrial membrane potential [22, 39]. These studies proved the caspase-9 pathway apoptotic induction by honey. Another research investigated that crude honey was sole responsible to induce apoptosis in human colon cancer and glioma C6 cell lines by elevating caspase-3 activation level and PARP (*Poly (ADP-ribose) polymerase*) cleavage. This characteristic was attributed to higher tryptophan and phenolic contents of honey [40, 145, 148]. Researchers showed that it induces apoptosis by upregulating and modulating the expression of pro- and antiapoptotic proteins in colon cancer cell lines HCT-15 and HT-29. It was found to elevate the expression of caspase-3, p53, and proapoptotic protein Bax. It downregulated the expression of antiapoptotic protein Bcl-2. The whole mechanism explained that ROS generation by honey results in the activation of p53, which in turn modulates the expression of pro or antiapoptotic proteins like Bax and Bcl-2 [22]. Honey administered with *Aloe vera* was found to boost the expression of proapoptotic protein Bax and decrease antiapoptotic protein Bcl-2 expression in Wistar rats with W256 mammary carcinoma implants [147, 148]. Furthermore, two different studies demonstrated that honey exerts its cancer therapeutic and cancer preventive effects in multiple ways such as modulation of immune response by ameliorating haematological parameters and stimulation of the intrinsic/mitochondrial apoptotic pathway at serological and cancer tissue level. In these studies, honey was given by oral feeding to Sprague-Dawley rat model using different concentrations such as 0.2, 1.0, and 2.0 g/kg body weight. It ameliorated the intrinsic apoptotic pathway through upregulation of the expression of proapoptotic proteins such as caspase-9, APAF-1 (apoptotic protease activating factor 1), p53, IFN-*γ* (interferon gamma), and IFNGR1 (interferon gamma receptor 1). Concomitantly, honey was found to downregulate the expression of antiapoptotic proteins such as Bcl-xL (B-cell lymphoma extra large), TNF-*α*, COX-2, E2 (estrogen), and ESR1 (estrogen receptor 1) [149, 150]. It was also demonstrated that honey alone induces intrinsic or caspase-9 apoptotic pathway with no evidence of the involvement of extrinsic or caspase-8 pathway [149, 150]. Flavonoids and phenolic contents of honey have been encountered to occlude the cell cycle of glioma [145], melanoma [146], colon [40], and cancer cell lines in G0/G1 phase. This inhibitory effect on tumor cell proliferation follows the downregulation of many cellular pathways via tyrosine cyclooxygenase, ornithine decarboxylase, and kinase [40, 145, 146, 151]. The mechanisms of action of honey include mainly its interference with multiple molecular targets and cell signaling pathways such as apoptotic, antiproliferative or cell cycle arrest, anti-inflammatory, estrogenic modulatory, antimutagenic, insulin modulatory, angiogenesis modulatory, and immunomodulatory pathways [6, 17]. Reviews by Ahmed et al. [6] and Erejuwa et al. [17] have well explained the possible mechanisms of anticancer effects of honey. Figures 10, 11, and 12 are showing a summarized presentation of mechanisms of anticancer effects of honey. Further studies are necessary to understand the exact influence of honey on the apoptotic pathways in cancer cells like the activation of caspase-8, p21, p38 MAPK (mitogen-associated protein kinase and p38 pathways), p-38 JNK (c-Jun N-terminal kinase), release of cytochrome c, and the suppression of antiapoptotic proteins such as IAP (inhibitor of apoptosis proteins), c-FLIP (cellular Flice inhibitory protein), and Akt (altered PI3 kinase), and the initiation of extrinsic pathway of apoptosis by induction of TRAIL (TNF-related apoptosis-inducing ligand) and Fas (fatty acid synthase-associated protein) receptor stimulation in cancer cells.

### 2.9. Antiproliferative Effects of Honey

Cell divides into two through cell cycle to replace cell death. The cell cycle comprises of three distinguished phases known as G0, G1, S, and G2/M. Cells remain still in G0 phase and not participating in the cell division. Cell gears up in G1 phase to move through cell division, and S phase involves synthesis of DNA. G2 and M phases are just ready to mitosis with 4n DNA. All the events in the cell cycle are regulated and monitored by several different proteins. The control panel of cell cycle comprises cyclins and cyclin-dependent kinases. G1/S phase transition is a vital regulatory point, where cell's fate is destined for quiescence, proliferation, differentiation, and apoptosis. Overexpression and dysregulation of cell cycle growth factors such as cyclin D1 and cyclin-dependent kinases (CDK) are linked with pathogenesis. The loss of this regulation is the hallmark of cancer as well [152]. The nuclear protein Ki-67 is a novel marker to probe the growth fraction of cell proliferation. It is absent in the resting phase (G0), but expressed during the cell cycle in all the proliferation phases (G1, S, G2, and mitosis) during cell cycle [153].

**Figure 13:**
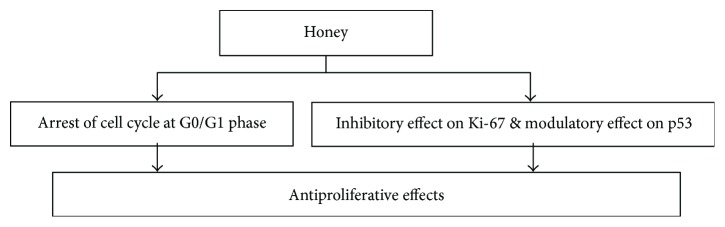
Mechanisms of antiproliferative effects of honey.

Administration of honey and *Aloe vera* solution showed a marked decrease in expression of Ki67-LI in tumor cells in Wistar rats having 256 carcinomas [147]. Honey and its several components like flavonoids and phenolics are reported to block the cell cycle of colon cancer cell lines in G0/G1 phase [40]. This inhibitory effect on tumor cell proliferation follows the downregulation of many cellular pathways via proteins such as tyrosine cyclooxygenase, ornithine decarboxylase, and kinase. Thus, it can be hypothesized that honey—or its components—mediates inhibition of cell growth and is due to perturbation in the cell cycle which may possibly lead to apoptosis [40, 145, 146, 154]. The cell cycle is a process regulated also by p53 protein, which as a result of DNA damage increases the levels of cyclin-dependent kinase (Cdk) inhibitors such as p21, p16, and p27 proteins [22]. Honey is reported to be involved in modulation of p53 regulation in colon cancer cell lines [22].  Figure 13 is depicting the possible mechanisms of antiproliferative effects of honey. Honey can suppress and or block the abnormal division of cells by working at various points of cell cycle. This is still urging to investigate the effect of honey on the levels of cyclin-dependent kinases, complexes of cyclins, cyclin-dependent protein kinases, and cyclin-dependent kinase inhibitors such as p16, p21, and p27 proteins in cell cycle proliferation.

### 2.10. Immunomodulatory Effects of Honey

Immunomodulation is progression of altering an immune system in a constructive or else damaging style. Many biological and chemical blends have the ability to modify immune system [155]. Immunomodulatory cytokines such as TNF-*α*, IL-1, IL-6, and IL-10 boost activation and proliferation of blood cells to induce phagocytic and lymphocytic activity, triggering an immunomodulatory response [156]. Honey was found to provoke stimulation to the immune system of the body to combat infections in rats. It stimulates T-lymphocytes, B-lymphocytes, and neutrophils in cell culture. B-lymphocytes ultimately stimulate the production of antibodies in primary and secondary immune responses against thymus-dependent and thymus-independent antigens [157]. It stimulates monocytes to release the cytokines such as TNF-*α*, IL-1, and IL-6, activating numerous aspects of immune response. Stimulatory action of honey towards leucocytes illustrates another action called “respiratory burst.” In this action, glucose of honey is absorbed to produce H_2_O_2_, which is considered as a leading constituent to stimulate the immune system. It also delivers substrate to glycolysis to produce energy in macrophages to allow them to perform immune modulatory function [4, 158].

Research has manifested that sugars which are slowly absorbed result in the formation of short chain fatty acid (SCFA) fermentation products. It is a probable mechanism that the ingestion of honey may result in SCFA formation. It has been established that either directly or indirectly, SCFA has immune modulatory actions. Thus, honey may stimulate the immune system via these fermentable sugars [159]. A sugar, nigerose, present in honey has been found to be immune protective [160]. Nonsugar components of honey may also be responsible for immunomodulation. Antioxidant content of the honey contributes to immunomodulatory action as well. Though antioxidant compounds have been reported to stimulate immune function *in vitro*, but there are no direct studies manifesting the effects of honey antioxidants on immune system [159, 160]. Different studies presented that Manuka, Pasture, Nigerian Jungle, and royal jelly honeys used in variant concentrations were found to increase IL-1*β*, IL-6, and TNF-*α,* apalbumin 1, production in cell line models [35, 78, 161]. The active component in Manuka was 5.8 kDa, which increased the production of these cytokines and TNF-*α* via TLR4 (toll-like receptor-4) in cell line culture. These authors settled that the compound was not an amino acid, lipopolysaccharide, mineral, or vitamin urging probe to elucidate the nature of this immune regulatory compound [78]. Treatment with honey (0.2, 1.0, and 2.0 kg/kg) showed a potentiating effect on haematological parameters such as Hb, RBC, PCV, lymphocytes, and eosinophils. It also showed an increasing effect on IFN-*γ* and IFNGR1 at serum and cancer tissue level in rats induced with breast cancer [149, 150]. Honey, when tested using concentration 1.2 g/kg body weight, was found to increase the antioxidant agents (vitamin C and *β*-carotene), monocytes, lymphocytes, eosinophils, serum iron and copper, glutathione reductase, and trace elements (Zn and Mg) in healthy human subjects. It caused to decrease immunoglobulin E, ferritin, and liver and muscle enzymes, aspartate transaminase, alanine transaminase, lactate dehydrogenase, creatinine kinase, and fasting blood sugars [56].

**Figure 14:**
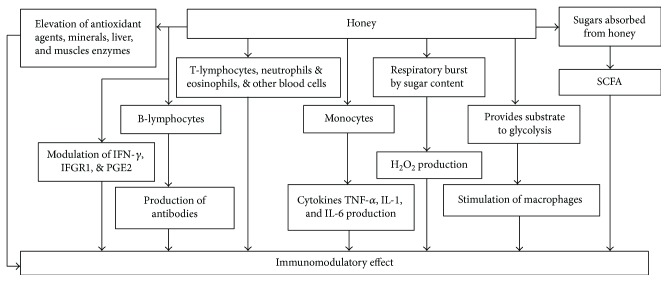
Mechanisms of immunomodulatory effects of honey. IFN-*γ* = interferon gamma; IFNGR1 = interferon gamma receptor 1; IL = interleukin; TNF-*α =* tumour necrosis factor alpha; PGE2 = prostaglandin E2; SCFA = short chain fatty acid.

The results of clinical trials showed that Life Mel honey (LMH) reduced the incidence of anaemia in 64% of patients by decreasing thrombocytopenia and neutropenia [162]. A study demonstrated that probiotic bacteria in honey have multiple actions in immunity: (a) protect the damaged immune system; (b) enhance the levels of circulating immunoglobulins, frequency of interferon and immunophagocytic activity; and (c) shift the events of the chemically induced reactions [163]. Synthetic medicines and natural products such as honey are supposed to inhibit PG production [164]. Immune function can be restored by the treatment with prostaglandin inhibitors or by reducing systemic PGE2 levels. The use of honey as a PG inhibitor to prevent a disease is emerging. Honey has shown inhibitory effects on PGE2 in carrageenan-induced acute paw edema in rats [107].  Figure 14 is showing the possible mechanisms of action of honey for its immune regulatory effects. Further probes are recommended to elucidate the effects and mechanisms of immune modulatory effects, perhaps using artificial immune challenges.

### 2.11. Cardiovascular Effects of Honey

Honey has the ability to regulate some cardiovascular risk factors which include blood glucose, cholesterol, CRP (C-reactive proteins), and body weight [43]. Honey contains glucose, fructose, and some trace elements such as copper and zinc, which may play a vital role to ameliorate the cardiac risks. It causes to decrease LDL (low-density lipoprotein), high-density lipoprotein cholesterol (HDL-C), triacylglycerole, body fat, glucose, and cholesterol levels in cardiac patients and healthy human subjects who took honey 70 g for 30 days. It retards the level of CRP, which stimulates nitric oxide production [43]. Nitric oxide has many cardioprotective effects which include regulation of blood pressure, vascular tone, inhibition of platelet aggregation, leukocyte adhesion, and prevention of smooth muscle cell proliferation [165]. NO acts as a critical mediator for vasodilation in blood vessels. It is induced by many factors such as acetylcholine, shear stress, and cytokines via eNOS synthesis. NO causes phosphorylation of several proteins that causes smooth muscle relaxation. The vasodilatory effect of NO plays a major role in renal regulation of extracellular fluid homeostasis and is also critical for the regulation of blood pressure and blood flow [165, 166]. Some flavonoids in honey have been reported to modulate cardiovascular risks by decreasing oxidative stress and increasing nitric oxide (NO) bioavailability. Similarly, rutin promotes NO production by enhancing eNOS gene expression and its activity. Naringin inhibits hypercholesterolemia-induced intercellular adhesion molecule-1 (ICAM-1) expression on endothelial cells. Recent studies have shown that catechin and quercetin as major honey flavonoids have inhibitory effects on the development of aortic atherosclerotic lesions and atherogenic modification of LDL [167].

**Figure 15:**
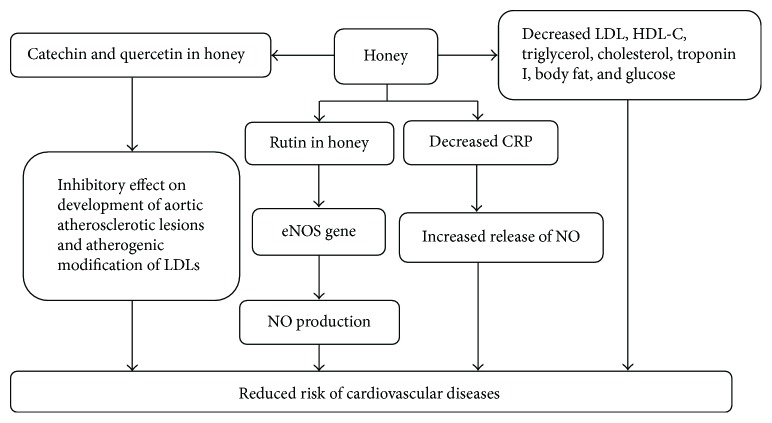
Mechanisms of cardiovascular protective effects of honey. eNOS = endothelial nitric oxide synthase; NO = nitric oxide; LDL = low-density lipoprotein; HDL-C = high-density lipoprotein cholesterol; CRP = C-reactive proteins.

Honey pretreatment was found to restore the decreased levels of enzymes such as superoxide dismutase, glutathione peroxidase, and glutathione reductase including creatine kinase-MB, lactate dehydrogenase, aspartate transaminase, and alanine transaminase against isoproterenol-induced myocardial infarction in Wistar rats [168]. It shows that honey gives defense from harmful effects prompted by free lethal radicals [169]. Another study has shown that honey causes to increase antioxidant markers in rat myocardial infraction model ameliorating cardiac troponin I (cTnI), triglycerides (TG), total cholesterol (TC), and lipid peroxidation (LPO) products [169]. All the possible mechanisms of cardiovascular effects of honey have been demonstrated in Figure 15. However, the exact mechanisms of action of honey still remain obscure for its cardiovascular effects. This urges for further investigation.

## 3. Pharmacokinetics of Honey

Literature lacks reports for the pharmacokinetics of honey. However, research has shown that honey may affect the pharmacokinetics of some drugs [170]. In vivo human studies reported that honey interferes with the activity of cytochrome p450 (CYP450) isozymes [170]. Preliminary clinical investigations for the effect of honey on CYP450 activity suggest that honey might increase CYP3A4 activity; however, it does not affect the activity of CYP2D6 and/or CYP2C19. It was also observed that increased CYP3A4 activity requires regular ingestion of honey, while occasional ingestion is unlikely to significantly affect drug plasma concentrations. Thus, honey may cause altered response to drugs metabolized by CYP3A4 [170]. CYP3A4 is the major phase I drug-metabolizing enzyme, and P-glycoprotein is an ATP-dependent drug efflux pump that regulates the intestinal absorption of orally administered drugs. In contrast, another human study reported that daily consumption of honey does not affect hepatic and intestinal CYP3A and P-glycoprotein activities [171, 172].

## 4. Limitations of Honey

Honey should be evaluated for its toxicological effects based on plants and or nectar source. Though not all, but intoxication by honey may be expected, for instance, mad honey is contaminated with grayanotoxin. Grayanotoxin is found in rhododendron plants in countries such as China, Tibet, Turkey, Nepal, Myanmar, Japan, New Guinea, Philippines, Indonesia, and North America. Mad honey collected in spring is more toxic containing more grayanotoxin [173]. Grayanotoxin causes intoxication which may include weakness, dizziness, excessive perspiration, hypersalivation, nausea, vomiting, and paresthesias. It may even lead to life-threatening cardiac complications such as complete atrioventricular block [173]. Honey may become contaminated with germs from plants, bees, and dust during production, collection, and/or processing. Fortunately, antimicrobial activity of honey ensures that most contaminating germs cannot survive or reproduce. However, bacteria that can reproduce using spores, including those that cause botulism, may survive. This is the reason that botulism has been reported in infants given honey orally. To solve this issue, honey or medical-grade honey should be irradiated to inactivate the bacterial spores [174]. Sometimes, food allergy due to honey is frequently accompanying with pollen allergy due to the presence of pollens during its collection. Thus, honey may have the possibility of sensitivity in any patient with suspected but unresolved food allergy [175]. A typical consumption of sugar and high fructose corn syrup (HFCS) totals the nearly ¾ pound per day for every individual above age 2. However, an amount, which simply overwhelms, results in elevated blood sugar levels, excessive insulin release, and resultant fat production and storage in the liver [176].

## 5. Conclusion

Honey can be considered a serine potential natural antioxidant medicine. Evidence-based research shows that honey acts through a modulatory road of multiple signaling pathways and molecular targets. It may interfere with multiple targets in cell signaling pathways such as induction of caspases in apoptosis, stimulation of TNF-*α*, IL-1*β*, IFN-*γ*, IFNGR1, p53, and immune cells, inhibition of cell proliferation, cell cycle arrest, inhibition of lipoprotein oxidation, IL-1, IL-10, COX-2, LOXs, and PGE2, and modulation of other diverse targets. This results in triggering the amelioration of antioxidant, antimutagenic, anti-inflammatory, immune regulatory, and estrogenic responses to abate different types of diseases. Effect of honey on pharmacokinetics of drug leads to dissimilar progressions of the body. Further research is needed to establish the possible mechanisms involved. More clinical and experimental trials are also intended to validate the authenticity of honey either alone or as an adjuvant therapy.

## Figures and Tables

**Table 1:** General composition of honey [50, 51].

Component	Value/100 g
Total carbohydrates	82.4 g
Fructose	38.5 g
Glucose	31.28 g
Sucrose	1.31 g
Maltose	7.31 g
Total acid as gluconic	0.57 g
Moisture content	17.1 g
Ash	0.169 g
Fibre	0.2 g
Amino acids/proteins	0.3 g
N	0.041 g
Fe	0.42 mg
K	52 mg
Ca	6.00 mg
P	4.00 mg
Mg	2.00 mg
Cu	1–100 *μ*g/g
Zn	0.22 mg
Vitamin B2	0.038 mg
Vitamin B3	0.21 mg
Vitamin B5	0.068 mg
Vitamin B6	0.024 mg
Vitamin B9	2 *μ*g
Vitamin C	0.5 mg
Miscellaneous groups	—
